# Dobutamine stress echocardiography for assessing the role of dynamic intraventricular obstruction in left ventricular ballooning syndrome

**DOI:** 10.1186/1476-7120-8-11

**Published:** 2010-04-09

**Authors:** Mario Previtali, Rita Camporotondo, Alessandra Repetto, Stefania Panigada

**Affiliations:** 1 From Department of Cardiology, IRCCS Fondazione Policlinico San Matteo, University of Pavia School of Medicine, Pavia, Italy

## Abstract

**Background:**

Dynamic intraventricular obstruction has been observed in patients with left ventricular ballooning syndrome (LVBS) and has been hypothesized as a possible mechanism of the syndrome. The aim of this study was to assess the prevalence and significance of dynamic intraventricular obstruction in patients with LVBS.

**Methods and Results:**

Dobutamine stress echocardiography was carried out in 22 patients with LVBS (82% apical), all women, aged 68 ± 9 years. At baseline 1 patient had a > 30 mmHg LV gradient; during stress a LV gradient > 30 mm Hg developed in 6/21 patients (28%) and was caused by systolic anterior motion of the mitral valve in the 3 patients with severe gradient (mean 116 ± 29 mmHg), who developed mitral regurgitation and impaired apical wall motion and by obstruction at mid-ventricular level in the other 3 with a moderate gradient (mean 46 ± 16 mmHg). Compared with patients without obstruction those with obstruction had a greater mean septal thickness (11.6 ± .6 vs 9.8. ± 3, p < .01), a higher prevalence of septal hypertrophy (71% vs 7%, p < .005) and a higher peak wall motion score index (1.62 ± .4 vs 1.08 ± .4, p < .01).

**Conclusion:**

Spontaneous or dobutamine-induced dynamic LV obstruction is documented in 32% of patients with LVBS, is correlated with the presence of septal hypertrophy and may play a role in the development of LVBS in this subset of patients. In those without septal hypertrophy a dynamic obstruction is rarely induced with dobutamine and is unlikely to be a major pathogenetic factor of the syndrome.

## 

Left ventricular ballooning syndrome (LVBS) is a recently described acute cardiac syndrome mimicking acute myocardial infarction, characterized by reversible regional apical or midventricular dysfunction whose pathogenetic mechanisms are as yet undefined [1-4]. Sympathetic stimulation and increased catecholamine release secondary to emotional stress leading to myocardial stunning are likely to play an important role [5,6]. Recently it has been hypothesized that in the presence of a localized proximal-mid septal hypertrophy, stress-related increased sympathetic tone may induce dynamic intraventricular obstruction, that in turn increases myocardial wall stress distal to the obstruction and decreases regional subendocardial perfusion leading to the development of akinesia and dilation in the apical region [7]. Dobutamine administration can induce a dynamic LV obstruction in a significant number of patients undergoing the stress testing [8-11] and could therefore reproduce the spontaneous event in patients with LVABS. We therefore carried out dobutamine stress echocardiography in a group of patients with LVBS to assess the prevalence and pathogenetic role of dynamic intraventricular obstruction in LVBS.

## Methods

### Study population

The study population was composed of 22 consecutive patients admitted with an acute coronary syndrome who fulfilled the diagnostic criteria for LVBS proposed by Mayo Clinic group [4] including: 1) Chest pain associated with new ST-segment elevation or T wave inversion in ≥ 2 contiguous leads; 2) Absence of significant (≥ 50%)obstructive coronary artery disease and of an acute plaque rupture at coronary angiography carried out within 48 hours from onset of symptoms; 3) Transient reversible akinesia or dyskinesia and dilatation involving either the midventricular and apical segments with basal LV hyperkinesia (apical ballooning) or the midventricular segments with normal or hyperkinetic basal and apical segments (midventricular variant); 4) No evidence of recent major head trauma, intracranial bleeding, pheochromocytoma, myocarditis or hypertrophic cardiomyopathy. All patients were included in the Italian Multicenter Registry on Takotsubo syndrome.

### Dobutamine stress echocardiography

The test was carried out at a mean of 9 ± 14 days from admission. Beta-blocking therapy was withdrawn 24 hours before the test. At baseline LV wall thickness and dimensions were measured by M-mode echocardiography in parastemal long axis view according to standard methods; a ≥ 12 mm end-diastolic wall thickness was considered indicative of wall hypertrophy. Dobutamine was administered at an initial dose of 5 mcg/kg/min for 5 minutes, with increase to 10 and 20, 30 and 40 mcg/kg/min for 5 minutes each under electrocardiographic and echocardiographic monitoring. The peak LV velocity was measured at rest, at the end of each stage and at peak stress by continuous wave Doppler from apical 4-or 5 chamber view. A pressure gradient >30 mmHg with a late peaking was considered significant for a dynamic intraventricular obstruction [1]; the site of LV obstruction was identified by pulsed and color Doppler; systolic anterior motion of the anterior mitral leaflet was searched for in multiple views. LV end-diastolic and end-systolic volume using the area-length method and ejection fraction were measured in the apical 4 chamber view at baseline and at peak stress. Digitized basal, low dose, peak dose and recovery images were displayed in a quad screen format for off-line comparison. Regional wall motion was assessed on a 16-segment LV model [12] as previously described and a wall motion score index was calculated at baseline and at peak stress [13]. A new or worsening wall motion abnormality compared to baseline was considered diagnostic of myocardial ischemia. Myocardial viability was judged to be present in basally hypokinetic or akinetic segments when normalization or improvement in myocardial thickening and motion was observed in at least 2 contiguous segments after dobutamine. The test was interrupted according to previously described criteria [13]. At the end of the test intravenous propranolol (1-5 mg) was administered. All patients gave their informed consent to the test.

### Coronary Angiography

Left ventriculography and multiplane coronary angiography were carried out according to standard methods. Ejection fraction was calculated by the area-length method. Coronary artery disease was defined as a ≥ 50% reduction in the luminal diameter of a major coronary artery. The TIMI flow grade classification and TIMI frame count were used to assess coronary blood flow in the 3 coronary arteries and calculated as previously described [14]. A TIMI frame count ≤ 27 frames was considered normal. The study complied with the Declaration of Helsinki on the research on humans; the study protocol was approved by the local ethical committee. All patients gave written informed consent.

### Statistical analysis

Data are presented as mean ± SD. Continuous variables were compared using a paired or unpaired Student's t test; categorical variables were compared by Fischer exact test.

## Results

### Clinical findings

The main clinical findings of the patients studied are shown in table 1. All patients were women with a mean age of 68 ± 9 years (range 54-84). No patient had a history of angina. Presenting symptoms were chest pain in 21 patients, dyspnea in 2 and dizziness or syncope in 4. Coronary angiography showed normal coronary arteries in 14 patients (64%) and <50% stenosis of 1 vessel in 8 (36%); TIMI frame count in the left anterior descending, left circumflex and right coronary arteries was abnormally prolonged in 32, 32 and 36% of patients respectively. Mean LV ejection fraction was 48 ± 8%; 10/22 patients (45%) had an ejection fraction <50%. LV angiography showed a typical apical ballooning pattem in 18 patients (82%) and a midventricular ballooning with sparing of the apical segments in 4 (18%). In the acute phase a dynamic intraventricular gradient was documented in 3 patients (14%).

**Table 1:** Clinical characteristics of the patients studied

**Age (yrs)**	68 ± 9(range 54-84)
**Sex (M/W)**	0/22
**Hypertension**	17 (77%)
**Dyslipidemia**	11 (50%)
**Family history of CAD**	8 (36%)
**Diabetes mellitus**	1 (4%)
**Smoking**	1 (4%)
**Previous angina**	0
**Triggering event**	11 (50%)
**ECG in the acute phase:**	
ST-segment elevation	13 (59%)
Negative T waves	7 (32%)
Minor ST-T abnormalities	2 (9%)
**Coronary angiography**	
Normal coronary arteries	14 (64%)
<50% stenosis 1 vessel	8 (32%)
**Ejection fraction**	48 ± 8%

Abbreviations: M = men; W = women; CAD = Coronary artery disease.

### Dobutamine stress echocardiography

**Figure 1:**
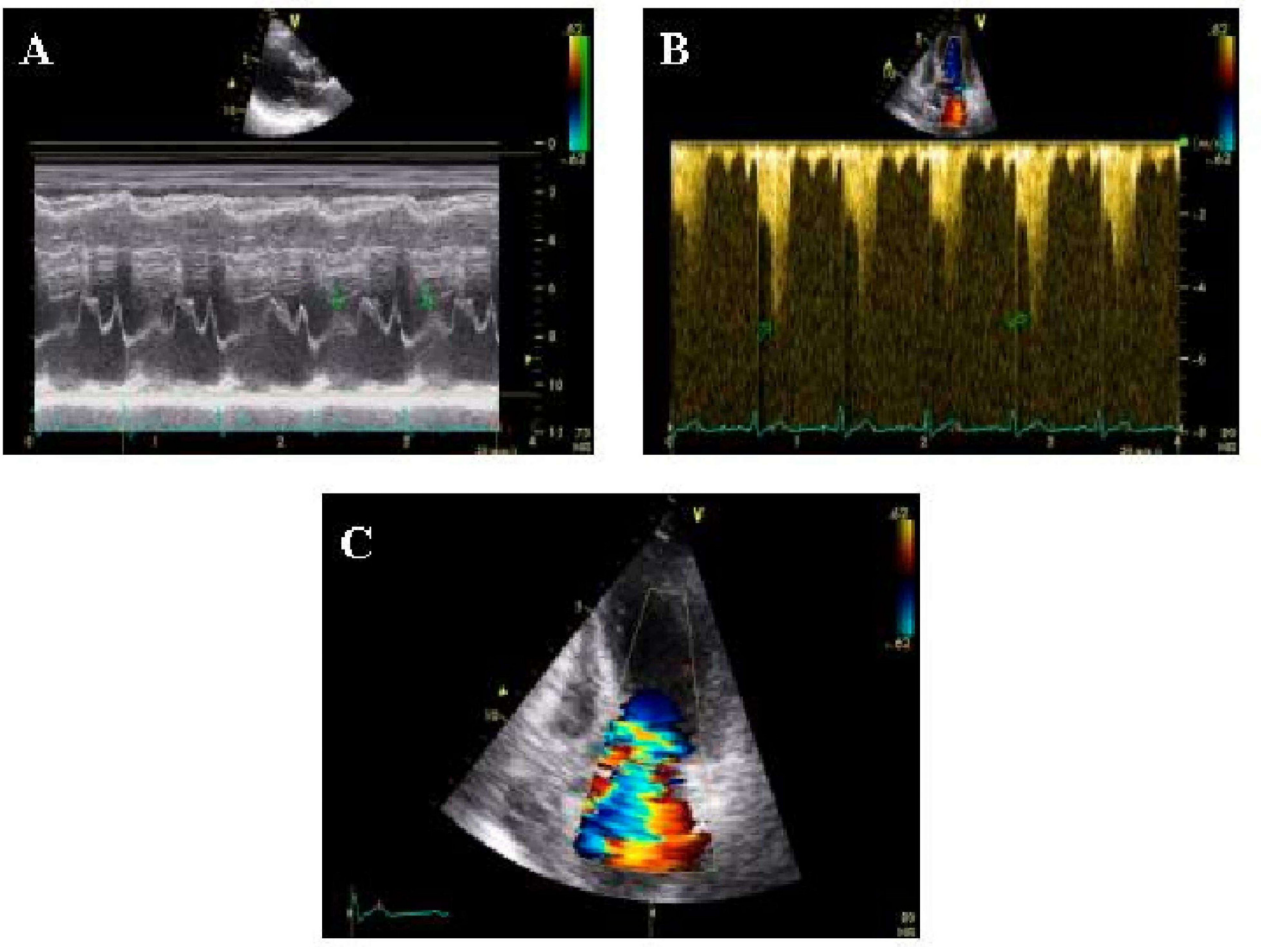
**Severe dobutamine induced-obstruction**. Severe dobutamine-induced dynamic LV obstruction (peak velocity 5 m/sec corresponding to a gradient of 100 mm Hg, panel B) caused by systolic anterior motion of the mitral valve (panel A) and associated with severe mitral regurgitation (panel C) in a patient with apical ballooning.

**Figure 2:**
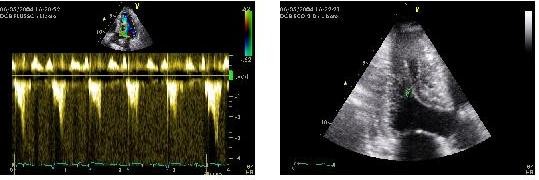
**Moderate dobutamine-induced obstruction**. Moderate dobutamine-induced dynamic LV obstruction (peak velocity 3 m/sec corresponding to a gradient of 36 mm Hg) caused by the iuxtaposition of the posterior papillary muscle to the hypertrophic septum in another patient with apical ballooning.

In basal conditions 5 patients showed a complete recovery of regional function while apical wall motion abnormalities were still present in 17. A localized proximal-mid septal hypertrophy was found in 6/22 patients (27%); no patient had a concentric LV hypertrophy. One patient showed a dynamic intraventricular gradient of 150 mmHg associated with systolic anterior motion of the mitral valve and mitral regurgitation and did not undergo DSE. In the remaining 21 patients heart rate increased from 67 ± 10 at baseline to 106 ± 18 beats/min at peak stress (p < .001) and systolic blood pressure increased from l30 ± 30 to 133 ± 27 mmHg (NS). Due to reduced diastolic filling time LV end-diastolic volume decreased from 99 ± 27 at baseline to 74 ± 30 ml at peak stress (p < .001) and end-systolic volume decreased from 48 ± 15 to 39 ± 23 ml (p < .002); mean walI motion score index improved from 1.4 ± .3 to 1.2 ± .4 (p < .01). A dynamic intraventricular gradient > 30 mmHg (mean 81 ± 44 mmHg, range 150-35 mmHg) with a late peaking developed in 6/21 patients (28%); the obstruction was localized in the outflow tract and caused by systolic anterior motion of the anterior mitral leaflet in 3 patients (mean gradient 116 ± 29 mmHg) (Figure 1) and at papillary muscle level in the other 3 (mean gradient 46 ± 16 mmHg) (Figure 2). The changes in regional wall motion in relation to dobutamine-induced obstruction are shown in Table 2. Of the 2 patients with a mild to moderate obstruction (gradient < 40 mmHg), both at papillary muscle level, 1 had normal baseline regional function and showed no change with dobutamine and 1 showed a partial recovery of regional wall motion abnormalities. Of the 4 patients with a severe obstruction (gradient > 40 mm Hg) 3 showed a biphasic response (improvement at low dobutamine doses followed by impaired apical wall motion associated with the development of obstruction) and 1 a direct deterioration of function in the apical region. In 3/4 of these patients the obstruction was localized in the outflow tract and associated with severe mitral regurgitation secondary to systolic anterior motion of the mitral valve (Additional files 1 and 2). No correlation was found between stenosis of left anterior descending coronary artery and stress echo positivity. On the other hand, of the 15 patients without obstruction, 6 showed a complete recovery and 6 a partial recovery of baseline regional wall motion abnormalities, 1 had no significant change and 2 had a normal regional function already at baseline. During the test > l mm ST-segment depression occurred in 1/6 (17%) patients with and in 0/15 without obstruction and positive T waves in the precordial leads developed in 3/6 patients with and in 4/15 without obstruction. No patients complained of chest pain or dyspnea during the test. In all cases dynamic obstruction and regional wall motion abnormalities rapidly disappeared after intravenous propranol. No major complication occurred during the test. Both patients with and those without contractile reserve during the test showed a complete recovery of regional function during follow-up.

**Table 2:** Changes in apical wall motion in relation to dobutamine-induced obstruction in the population studied.

	Dynamic obstruction (n = 6)	No dynamic obstruction (n = 15)
**Biphasic response**	3	0
**Direct impairment**	1	0
**No changes**	1	1
**Partial improvement**	1	6
**Complete normalization**	0	6
**Normal baseline wall motion**	0	2

### Comparison or patients with and without obstruction

Table 3 compares the clinical, angiographic and stress echocardiographic findings of the 7 patients with spontaneous or dobutamine induced obstruction with those of the 15 without obstruction. The main clinical characteristics, baseline and peak stress heart rate, blood pressure, LV volumes and ejection fraction were not significantly different between the 2 groups. Compared with patients without obstruction those with obstruction had a significantly greater mean septal thickness (11.6 ± .6 vs 9.8 ± .3, p < .01) and a higher prevalence of localized septal hypertrophy (71% vs 7%, p < .005). Despite similar baseline wall motion score index, patients with a dynamic gradient had a significantly higher wall motion score index at peak stress (1.62 ± 4 vs 1.08 ± .4, p < 0.01) indicating an impairment in regional wall motion associated with the obstruction. In relation to the site of LV ballooning, a dynamic obstruction was documented in 6/18 patients with apical and in 1/4 with midventricular ballooning (33% vs 25%, NS).

**Table 3:** Comparison of patients with (group 1) and without (group 2) dynamic intraventricular obstruction.

	Group 1	Group 2	p-value
**Age (years)**	67 ± 9	68 ± 9	ns
**Hypertension (%)**	85	73	ns
**IVS thickness (mm)**	11.6 ± .6	9.8 ± .3	<0.01
**PW thickness (mm)**	9.8 ± .6	8.7 ± .1	ns
**Septal hypertrophy (%)**	71	7	<0.01
**BPs basal (mmHg)**	131 ± 29	129 ± 27	ns
**BPd basal (mmHg)**	74 ± 10	69 ± 14	ns
**HR basal (b/min)**	70 ± 8	66 ± 12	ns
**HR dobutamine (b/min)**	101 ± 18	108 ± 18	ns
**LVEDV basal (ml)**	99 ± 29	99 ± 28	ns
**LVEDV dobutamine (ml)**	81 ± 29	72 ± 32	ns
**LVESV basal (ml)**	44 ± 19	48 ± 14	ns
**LVESV dobutamine (ml)**	34 ± 23	40 ± 24	ns
**LVEF basal (%)**	54 ± 5	48 ± 14	ns
**LVEF dobutamine (%)**	61 ± 16	67 ± 7	ns
**WMSI basal**	1.44 ± .4	1.29 ± .4	ns
**WMSI dobutamine**	1.62 ± .4	1.08 ± .4	<0.01

The patient with dynamic obstruction in basal conditions is included in group 1.

Abbreviations: IVS = interventricular septum, PW = Posterior wall; BPs = Systolic blood pressure; BPd = Diastolic blood pressure; HR = Heart rate; LVEDV = Left ventricular end-diastolic volume; LVESV = Left ventricular end systolic volume; LVEF = Left ventricular ejection fraction; WMSI = Wall motion score index.

## Discussion

### Prevalence and significance of dynamic LV obstruction in LVBS

Many pathophysiological mechanisms, including direct toxic effects of catecholamine overflow, epicardial coronary artery spasm and diffuse coronary microvascular dysfunction, have been hypothesized as possible causes of LVBS, but none of them has been convincingly demonstrated [4]. The close temporal relation with psychological stress and increased plasma levels of catecholamines documented in some studies suggest that sympathetic stimulation and toxic effects of catecholamines may play a major role [5,6], however, other studies have documented no significant elevation in plasma catecholamine and cortisol levels [15]. Bybee et al found an increased TIMI frame count in all major coronary arteries of patients with LVBS [16]; other studies showed a transient impairment of coronary flow reserve in the acute phase with early recovery paralleling the recovery of wall motion abnormalities [17,18]. These findings suggest that coronary microvascular dysfunction may play a pathogenetic role, but it has not been as yet clarified whether microvascular dysfunction is a primary or secondary phenomenon. Coronary epicardial spasm has been documented in a significant proportion of Japanese patients [1,2], but is rare in Caucasian patients [3-6,19]. During the acute phase a transient dynamic intraventricular gradient was documented in 18% of patients in the largest population of LVBS so far reported [1]; in other smaller studies the prevalence of dynamic obstruction in the acute phase ranged from 12.5% [16] to 23% [5]. These findings and the association of dobutamine-induced dynamic intraventricular gradient with worsening of apical wall motion recently reported in 2 patients with LVBS and septal hypertrophy [7] suggested a possible pathogenetic role for dynamic intraventricular obstruction in LVBS. The prevalence of dobutamine-induced dynamic obstruction in our patients with LVBS is similar to that reported by previous studies in patients undergoing dobutamine stress echocardiography for evaluation of coronary artery disease [8-11,20-22], that showed a prevalence ranging from 13% in the study by Heinle et al [10] to 53% by Wagner et al [9]. Therefore, the development of a mild to moderate intraventricular gradient during dobutamine stress is frequent and may have no major clinical significance in the majority of patients with LVBS as in the general population. The significant association found in our patients between dynamic intraventricular gradient and septal hypertrophy is in keeping with the studies by Khanal et al [21] and Wagner et al [9], who showed that dobutamine-induced LV cavity obliteration was associated with female sex and LV hypertrophy. Thus, the dynamic intraventricular obstruction documented in our patients does not seem to be a specific feature of LVBS, but rather depends upon the presence of a localized septal hypertrophy that is frequently found in elderly women. On the other hand, in a minority of patients with LVBS a severe dynamic intraventricular obstruction can play a role in the development of LVBS by increased myocardial wall stress and decreased subendocardial perfusion in the apical region leading to myocardial ischemia on a hemodynamic basis; this hypothesis is supported by the fact that all 4 patients with severe dobutamine-induced obstruction showed a significant impairment in apical wall motion. The hypothesis of a coronary spasm during dobutamine stress or at the time of beta-blocking infusion as the mechanism of the wall motion abnormalities observed in these patients is unlikely because none of these patients developed ST-segment elevation, which is the hallmark of coronary artery spasm and the wall motion changes occurred before administration of beta-blockers and were abolished by them. On the basis of our data, it is not possible to assess whether dynamic LV obstruction plays a primary pathogenetic role, leading to the development of an apical ballooning as suggested by Merli et al [7], or is secondary to the distortion of the LV geometry caused by apical akinesia and basal hyperkinesia and acts as a perpetuating mechanism of the ballooning. Another important finding of the study is the association of a major dynamic obstruction with the development of severe mitral regurgitation due to systolic anterior motion of the anterior mitral leaflet in 15% of patients with LVBS. Severe mitral regurgitation increases pulmonary capillary pressure and decreases LV forward stroke volume and may therefore be an important yet unrecognized mechanism contributing to acute LV failure and shock reported in 20% to 40% of patients with LVBS [1,4,17]. Acute mitral regurgitation due either to systolic anterior motion of the anterior mitral leaflet or to displacement of the papillary muscle with impaired leaflet coaptation has been recently reported in 20% of patients with LVBS and has been associated with a worse prognosis [23]. On the other hand, our study demonstrates the absence of a spontaneous or dobutamine-induced dynamic LV gradient in >65% of patients with LVBS; this finding suggests that in the majority of patients with LVBS the development of the syndrome is not related to a dynamic intraventricular obstruction elicited by increased sympathetic tone.

### Clinical implications

Dobutamine stress echocardiography may be a useful tool for assessing the presence and significance of LV dynamic obstruction in patients with LVBS and for clinical decision making. However, the safety of the test in this setting has not been as yet assessed and further studies on a larger number of patients are warranted. Beta-blocking drugs decrease LV contractility and can prevent the development of dynamic intraventricular gradient and should therefore be the treatment of choice for patients with dobutamine-induced dynamic LV obstruction. The subgroup of patients who develop a severe outflow obstruction associated with major mitral regurgitation are at a higher risk of acute LV failure in the case of recurrences and should benefit from chronic beta-blocking therapy at maximal tolerated doses. and from intravenous beta-blockade if episodes of apical ballooning with dynamic obstruction recur.

## Competing interests

The authors declare that they have no competing interests.

## Authors' contributions

MP planned the study, performed and interpreted the echocardiographic stress tests and wrote the manuscript; RC collected the clinical data, performed and interpreted the echocardiographic stress tests and reviewed the manuscript**; **AR performed the angiographic studies and reviewed the manuscript; SP collected the clinical data, performed the echocardiographic stress tests and reviewed the manuscript. All the authors have red and approved the final manuscript.

## Supplementary Material

Additional file 1 **Example of dobutamine-induced dynamic obstruction in one of the patient studied**. Apical 4-chamber view in basal conditions showing no evidence of dynamic obstruction and mitral regurgitation and normal apical wall motion. Click here for file

Additional file 2 **Apical 4-chamber view during dobutamine stress showing severe mitral regurgitation due to mitral SAM and impaired apical wall motion**. Click here for file
